# Upscaling of Copper Slag-Based Geopolymer to 3D Printing Technology

**DOI:** 10.3390/ma17225581

**Published:** 2024-11-15

**Authors:** Barbara Kozub, Mateusz Sitarz, Szymon Gądek, Celina Ziejewska, Katarzyna Mróz, Izabela Hager

**Affiliations:** 1 Department of Materials Engineering, Faculty of Material Engineering and Physics, Cracow University of Technology, 37 Jana Pawła II Street, 31-864 Cracow, Poland; szymon.gadek@pk.edu.pl; 2 Chair of Building Materials Engineering, Faculty of Civil Engineering, Cracow University of Technology, 24 Warszawska Street, 31-155 Cracow, Poland; mateusz.sitarz@pk.edu.pl (M.S.); katarzyna.mroz@pk.edu.pl (K.M.); izabela.hager@pk.edu.pl (I.H.); 3 Department of Applied Computer Science, Faculty of Mechanical Engineering, Cracow University of Technology, Al. Jana Pawła II 37, 31-864 Cracow, Poland; celina.ziejewska@pk.edu.pl

**Keywords:** 3D printing, geopolymer, copper slag, forming in mold, mechanical properties

## Abstract

Additive manufacturing using cement has evolved rapidly in recent decades, revolutionizing the construction industry. This technology automates building structures through computer-aided design, offering benefits such as reduced material waste, optimized material distribution, and the ability to use composite materials. This paper aims to examine the potential of using copper-slag-based geopolymers in 3D printing. Geopolymers have gained popularity as an alternative and more energy-efficient material to traditional building materials, while copper slag allows for reducing and managing mining industry waste. Moreover, samples formed in molds based on the same material were produced to evaluate the method of manufacturing on the mechanical properties of geopolymers. This paper presents an evaluation of the mechanical properties including the compressive, flexural, and shear strength of the layered material. It reveals promising results, with strength development mainly observed within the first 14 days. The results show that the compressive strength after 28 days of curing is 46.4 MP and 42.1 MPa for formed and printed samples, respectively. Furthermore, the average bending strength value ranges between 7.4 MPa and 7.8 MPa, regardless of the bending direction and forming method. The obtained results show that printed geopolymers demonstrate adequate layer bonding, confirming the profitability of the 3D printing technology. This research confirms that 3D printing technology enables the use of geopolymer binder materials based on copper slag, which opens the door to sustainable alternatives in construction practices.

## 1. Introduction

Over the last few decades, 3D concrete printing technology, also known as additive manufacturing of concrete, has undergone rapid development. This technology is defined as “the process of combining materials to produce elements or objects from 3D model data, usually layer by layer, in opposite to subtractive manufacturing methods” [1,2,3]. The automation of the building process of structures based on 3D printing technology is an example of the use of computer-aided design software, from which a digitally saved model is sent to software that allows the model to be divided into layers, which is then sent to a printing device in the form of a digital recording. This technology is an innovative solution and is still in the stage of intensive development. More and more new solutions are appearing on the market, offering many advantages over traditional manufacturing methods. The advantages mentioned in the literature include primarily the reduction of material waste generated in the production process and an optimized material distribution process developed based on application requirements. In addition, 3D printing contributes to a significant reduction in construction time, allowing for an increase in the efficiency of construction works [1,4,5,6,7]. Moreover, additive manufacturing enables the fabrication of prefabricated elements off-site, as well as on-site production, which reduces energy consumption during production [8,9,10]. Furthermore, the use of this technology reduces labor requirements and lowers the number of workplace accidents in the construction industry [11]. In addition, 3D printing enables the implementation of complex structural designs and therefore increases the freedom in engineering design [12,13]. Finally, 3D printing allows for the use of composite materials, enhancing the quality and durability of the structure. However, there are also several limitations of 3D printing technology that limit its use in the construction industry. Currently, one of the biggest limitations, due to the lack of legal regulations, is the problem of the certification of products manufactured with this technology, which negatively affects the possibility of introducing mass production. Another issue is the cost of large format printers, which can exceed several million euros, as well as subsequent high maintenance costs. The quality of printed objects may also be questionable—some of the materials used may have low resistance to exposure to various environmental conditions or may present a low bearing capacity [14]. Great emphasis should also be placed on specifying the quality and repeatability of printouts [15].

As shown by Zhang et al. [16,17], the use of 3D printing technology in construction allows for reducing waste generated during production by up to 30 to 60%, production time by 50 to 70%, and equally important production costs by 50 to 80%. As a result, 3D concrete printing, although developing slower than in other sectors, including medicine, pharmacy, or even art, has attracted significant interest around the world in recent years. The first additive manufacturing method used in the construction sector, developed in the mid-1990s, was the Contour Crafting technology [18,19,20]. This technology is based on fused deposition modeling—the extrusion and deposition of 3D-printed concrete [21]. Other methods described in the literature are Concrete Printing [22] and D-shape [15,23].

Concrete Printing is a technology developed by a team from the University of Loughborough, which is based on the extrusion process. Due to dimensional limitations, it is mainly used for the production of basic prefabricated concrete elements [24]. Concrete Printing, compared to Contour Crafting, is characterized by a lower deposition resolution, which gives this method greater control when printing more complex shapes [22].

The D-shape printer is one of the largest 3D printers in the world, which uses building materials in powdered form in the printing process and connects them using an adhesive binder or a similar binding component. This technology allows the creation of elements of large dimensions and complex shapes [15,21].

In recent years, new proposals have also appeared, such as particle printing based on stereolithography technology, which involves the selective deposition of a liquid binder on a powder substrate, causing its particles to bind [5].

Currently, one of the most popular 3D printing methods is the extrusion method, in which the product is produced layer by layer from material pressed through a nozzle [5,6,25]. Furthermore, this printing technique is used for creating buildings onsite, often composed of complicated shapes [26]. Nonetheless, it is worth mentioning that it was estimated that over 40% of resources expended globally are used in the construction industry, whereas concrete is the most popular building material [27,28]. The composition of 3D-printed concretes contains Portland cement in an amount ranging from 15 to 45% of the total proportion of the mixtures, and it is a key ingredient [4,29]. However, as Mohan et al. write [30], it is worth paying attention to the fact that cement materials used in the 3D printing process in the construction industry are usually characterized by a higher binder content compared to traditional forming techniques. Unfortunately, the increasing consumption of Portland cement has potential consequences, such as increased material costs and a negative impact on the sustainable economy—the production of Portland cement is responsible for approximately 8% of CO_2_ emissions worldwide [31,32]. Therefore, recently, there has been more and more research focusing on the possibility of replacing Portland cement with environmentally friendly materials. One of the promising directions is the use of geopolymers, which not only have a lower negative impact on the environment but also exhibit many advantages, for instance, good fire resistance performance, high durability and corrosion resistance, and excellent mechanical characteristics, particularly great early compressive strength as compared to Portland cement [33,34,35]. Geopolymers find many applications nowadays due to their properties, for instance, as a material suitable for highway infrastructure [36], thermal insulation [37], immobilization of hazardous substances [38], fire-resistant coatings [39], and construction materials [40].

By definition, geopolymers fall into the same category as cement of the third generation made from limestone and standard Portland cement [41]. Geopolymers refer to amorphous synthetic alkaline aluminosilicates that fall within the category of inorganic polymers. They come into being as a consequence of a chemical reaction involving the elements silicon (Si), aluminum (Al), and minerals that are derived from the earth. The process of geopolymer synthesis is performed at temperatures in the range of 20 to 120 °C [42]. Although the chemical makeup of geopolymers is comparable to that of zeolites, geopolymers may be differentiated from zeolites by their amorphous microstructure. In the technological process, various naturally occurring materials are used as solid raw materials for the production of geopolymers. These naturally occurring materials include kaolinite, metakaolin, feldspar, rice husk ash, volcanic rock powders, wood ash with a high calcium content, and industrial solid residues such as fly ash, slag, and mine tailings, amongst others [41,43,44,45,46,47,48]. The aluminosilicate sources have the greatest effect on the geopolymer synthesis process. The activity of these sources depends on their chemical and mineral makeup, as well as the shape and amount of the glassy phase [41]. In addition, for the final geopolymer to be stable, the raw materials must have a low water demand, a high amorphous content, and a suitable amount of reactive glassy content, and they must readily release aluminum [49]. All these characteristics must be present. The raw material has a high concentration of aluminum and a low concentration of calcium, both of which contribute to the construction of a three-dimensional network. This network structure is unique to geopolymer materials and is essential to their functioning. However, there are no sources in the literature that would specify the precise quantity of the base material that must include the various elements and oxides.

Professor Joseph Davidovits developed geopolymers in the 1970s, and these materials have recently gained popularity as a promising alternative to traditional cement-based construction materials [50]. They have been recognized as a promising alternative to cement-based construction materials intended not only for classic applications but also for 3D printing, which allows for increasing the sustainable development of the construction industry [4]. Therefore, there is a significant number of studies focusing on the possibility of using geopolymers to create sustainable concretes produced in 3D printing technology using various techniques, such as extrusion [51,52,53] or particle bed printing [54,55]. The research being conducted opens up new possibilities for creating 3D-printed concrete that not only meets technical and strength requirements but also minimizes the negative impact on the environment.

The development of geopolymer materials for 3D printing is influenced by many factors, including the mix design, selection of appropriate printing parameters, and the incorporation of reinforcements to improve mechanical properties, which play a key role in structural applications. In their study, Mukhametkaliyev et al. [56] showed that the compressive strength of geopolymer concrete can be significantly increased by optimal mix designs and the incorporation of fibers. They also found that geopolymer concrete is suitable for structural applications due to the early development of compressive strength. In addition, the printability and mechanical performance of geopolymer composites can be improved by additives and reinforcements [57,58]. Nematollahi et al. [59] and Munir et al. [60] showed in their works that the incorporation of fibers such as polypropylene or aramid improves the flexural and tensile strength of 3D-printed geopolymer materials.

On the other hand, Lim et al. [61] and Archez et al. [62] in their works showed that interlayer bonding, which is essential for maintaining the structure of 3D-printed parts, can also be improved by carefully controlling the printing process and material composition. Regarding the 3D printing of geopolymers, it is also worth highlighting another important aspect, which is their rheological properties. In general, these materials usually exhibit thixotropic behavior, ensuring shape retention during the printing process. Therefore, achieving the right balance between flow stress and viscosity is crucial for successful 3D printing applications [63]. Marczyk et al. [64] in their research show that materials that behave similarly to non-Newtonian fluids may be more suitable for 3D printing after optimizing the mixture design.

Since, in the future, the selection of sustainable binders may play a key role in the development of 3D printing technology in the construction industry, this work focuses on the possibility of 3D printing of geopolymers based on copper slag. Moreover, formed samples based on the same material are produced to evaluate the method of manufacturing on the mechanical properties of geopolymers.

## 2. Materials, Sample Preparation, and Testing Methods

### 2.1. Materials

The base material used to prepare the geopolymer paste for 3D printing is copper slag Koranel (Metallo, Beerce, Belgium) [65]. Koranel is a granulated Fe silicate containing traces of heavy metals, derived from secondary copper smelting followed by evaporation of zinc in an immersed plasma to recover Zn and P. The choice of Koranel copper slag, which is an industrial by-product, fits into the issues of sustainable development and circular economy. Since it is a material that contains a significant amount of iron silicates and other oxides, it is ideally suited for the synthesis of geopolymers. In general, as mentioned in the introduction of the manuscript, the production of geopolymers is associated with lower CO_2_ emissions during their production compared to traditional Portland cement. Therefore, the desire to create more environmentally friendly building materials was another factor in the choice of Koranel. Used for investigation, copper slag is also a readily available material, and its use can be cost-effective compared to the extraction and processing of primary raw materials. The oxide composition of the base material, shown in Table 1, is examined on an EDX-7200 spectrometer (Shimadzu, Kyoto, Japan).

**Figure 1:**
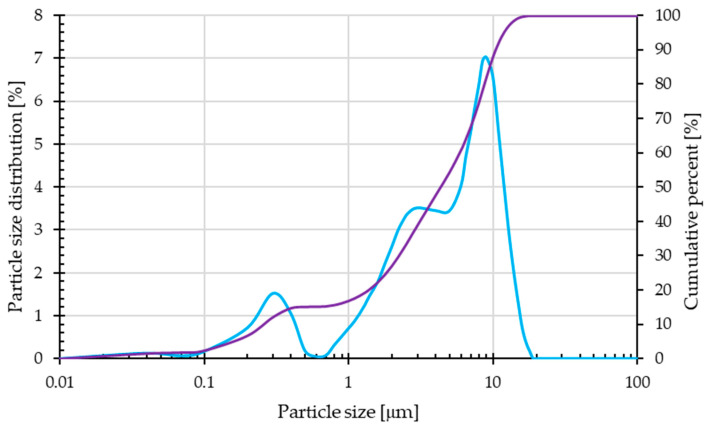
The particle size distribution curve (blue line) and the cumulative curve (violet line) of copper slag.

Figure 1 shows the particle size distribution and cumulative curve for the copper slag used. The results provided originate from a proprietary investigation conducted using the Anton Paar GmbH particle size analyzer located in Graz, Austria. Table 2 presents comprehensive data on the particle size distribution of copper slag, including key parameters such as the D_10_, D_50_, and D_90_ values; mean size; and span parameter.

The result of the copper slag particle size distribution analysis reveals a mean particle size of 5.34 μm with a standard deviation of 0.019 μm, which indicates a relatively uniform size distribution. The D_10_, D_50_, and D_90_ values are 0.26 μm, 4.33 μm, and 10.44 μm, respectively. The calculated span of 2.35 suggests a moderate breadth in the distribution of investigated material.

**Figure 2:**
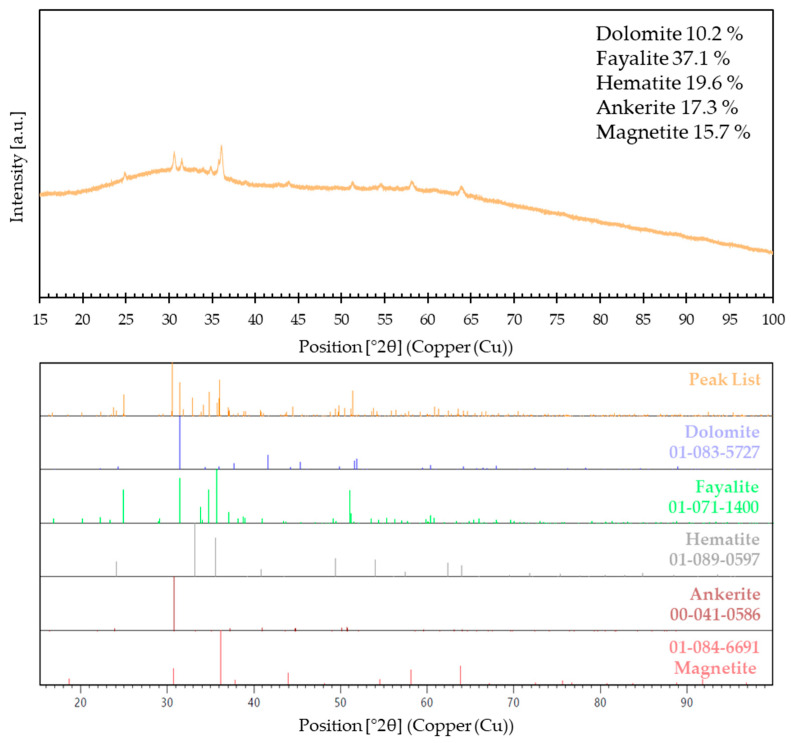
XRD patterns of Koranel copper slag.

The X-ray diffraction (XRD) pattern of Koranel, which is used as the base material in this research, is shown in Figure 2. The XRD analysis is performed on a PANalytical AERIS X-ray diffractometer (Malvern Panalytical, Almelo, The Netherlands) using Cu-Kα radiation with a scanning range from 10° to 100° 2θ and step size of 0.003° (2θ). Qualitative analysis of X-ray diffraction, performed using the ICDD PDF4+ database, allows the identification of the following phases: fayalite (card no.: 01-071-1400), dolomite (card no.: 01-083-5727), ankerite (card no.: 00-041-0586), magnetite (card no.: 01-084-6691), and hematite (card no.: 01-089-0597). Quantitative analysis carried out using the Rietveld method allows for determining the content of detected phases, the shares of which are 37.1%, 10.2%, 17.3%, 15.7%, and 19.6% for fayalite, dolomite, ankerite, magnetite, and hematite, respectively. However, these values should be taken as approximate due to the presence of an amorphous phase (as indicated by a broad hump in the range of 22 ° to 38° 2θ) and high background noise intensity.

Potassium silicate solution Geosil 14517 (Wöllner GmbH, Wöllnerstraße 26, 67065 Ludwigshafen, Germany) is used as the activator. The liquid to precursor ratio is 0.47. Aluminum metal powder Al-26.98 g/mol (Merck Life Science BV, Ildefonse Vandammestraat 5/7B, 1560 Hoeilaart, Belgium) and sodium oleate pure C_18_H_33_NaO_2_ (Merck Life Science BV, Ildefonse Vandammestraat 5/7B) are used as additional additives. The aluminum powder and sodium oleate pure were introduced in the amounts of 0.25% and 0.01% in relation to copper slag, respectively.

### 2.2. Printing Method and Setup

**Figure 3:**
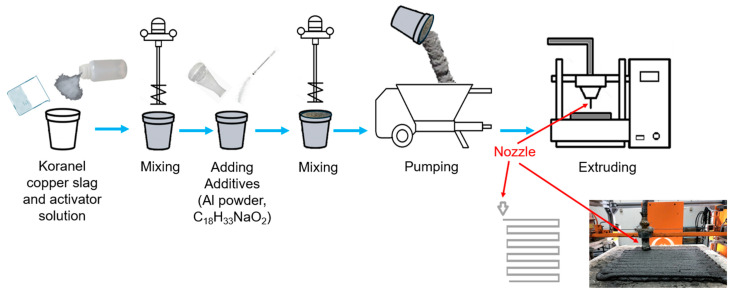
The schematic of the process for the 3D-printed sample preparation.

The geopolymer paste is prepared by mechanically mixing the dry ingredients with an alkaline solution. In the case of 3D printing, the prepared geopolymer paste is transferred to the pump, which then pushes the material to the printer. In the case of the formed samples made for comparative purposes, the finished geopolymer paste is transferred to the appropriate molds. Figure 3 presents the process of preparing the 3D printing samples in a schematic way.

**Figure 4:**
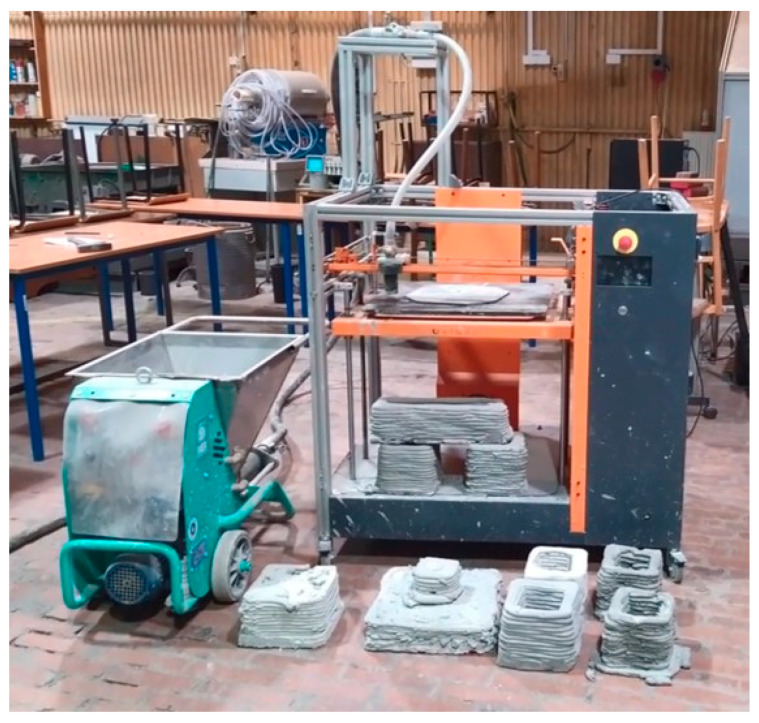
Instrumentation used during 3D printing of samples based on copper slag.

**Figure 5:**
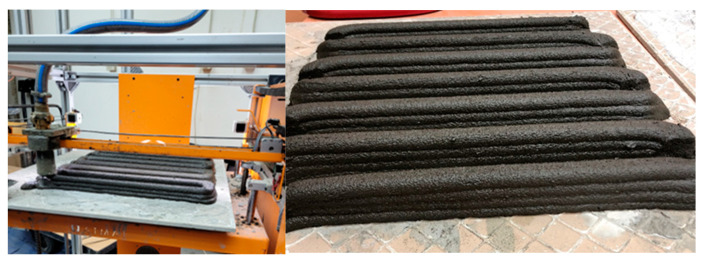
Prints made of geopolymer binder based on copper slag.

Figure 4 shows the tooling used during 3D printing. From the left, the Imer Small 50 device (Imer Group, Kraków, Poland) transports the material to the nozzles, which is equipped with a progressive cavitation pump, also known as the Moineau pump (the operating principle was patented in 1930). The device has a stainless-steel tank with a volume of 50 L. The inverter enables smooth adjustment of the material flow speed during printing, with the material feeding rate for printing the tested samples being approximately 2 dm^3^/min. The pump is connected directly through a hose transporting the material to the nozzle of the Galaxy printer—manufactured by ATMAT (Kraków, Poland). This device allows printing in FDM/FFF technology and has been appropriately adapted to print from concrete/geopolymer materials. The size of the printer’s worktable is 460 mm × 460 mm. When printing from the tested material, a nozzle with a diameter of Ø20 mm is used. Since the printer is not connected to the pump via an electrical system, the optimal printing method is Spiralize Outer Contour printing. Thanks to this, the material is transported continuously, and there is no problem with a lack of retraction. Printing from the tested material is performed by applying a 10 mm high layer of material (number of layers: 4) at a speed of 300 mm/s to a rough ceramic plate placed on the printing table. The printer nozzle moves in the XY plane, and the feed in the Z axis is achieved by changing the table height. After completing the printing process, the ceramic plate with the print is removed from the working field, while the print is left to harden under the foil at ambient temperature. A photo of the printout in the form of single lines is shown in Figure 5.

### 2.3. Experimental Methods and Testing Setup

This paper aims to compare the strength development of (i) traditionally formed (cast in standard molds) and (ii) printed material. The compressive and flexural strengths are tested for both types of specimens, after 5, 14, and 28 days. This made it possible to assess the influence of the printing method on any changes in strength development. Furthermore, the strength of the interlayer bonds in the printed specimens is analyzed by the direct tensile strength test and shear strength test. This research is carried out according to the procedure described by Hager et al. [66].

The layered structure of 3D-printed materials classifies them as orthotropic materials, resulting in a directional dependence of material properties [67]. This orthotropy results from the additive manufacturing process and highlights the importance of proper layer bonding for structural integrity. Notably, there is a lack of standardized testing for printed materials. Therefore, many research results are needed to develop guidelines and recommendations. The orthotropic nature of printed materials’ properties is a significant drawback in additive manufacturing structures. As a result, mechanical testing becomes imperative. Typically, strength tests are related to the direction of printing and include perpendicular, cross-sectional, and side evaluations.

The strength of the interlayer bond is a critical factor in the effectiveness of 3D printing technology. Assessing the bond strength requires selecting an appropriate method for measuring the bond between layers and understanding its behavior. Currently, the existing study of 3D-printed elements relies on methods used to measure the bond strength in materials such as concrete or wood [68]. Techniques used to evaluate the interlayer bond strength mostly include direct tensile, splitting, compression, flexure, and shear strength tests. In the literature, we can find different evaluations of this approach. In the opinion of Zhang et al. [69], the assessment of bond quality based on a compression test is sufficiently reliable. On the other hand, Nerella et al. [70] believe that such analyses should be approached with care. In the opinion of the authors, the analysis of interlayer connections based on static load tests is, for the purpose of assessing the mechanical strength of printed elements, more adequate. In a previous publication, the authors have provided a detailed description of the test method for 3D-printed elements [66].

**Figure 6:**
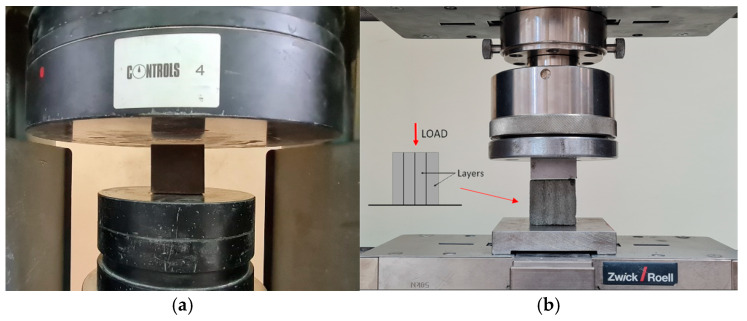
Test stand for compressive test: (**a**) formed samples, (**b**) printed samples.

The compressive strength test for the traditionally formed samples is carried out on cubes of 50 × 50 × 50 mm^3^. During the tests, the loading rate is 500 N/s. Figure 6a shows the test stand for compressive tests carried out with the Controls machine.

**Figure 7:**
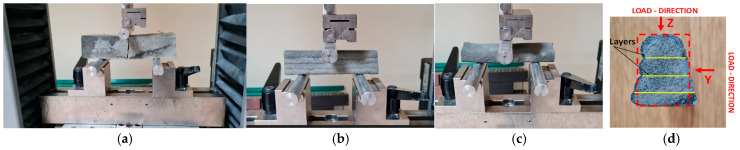
Bending strength testing: (**a**) formed samples, (**b**,**c**) printed samples, (**d**) load direction.

The printed samples are tested under compression in the direction longitudinal to the orientation of the layers. The example of its cross-section is presented in Figure 7d. The printed samples in the compression test have an area of approximately 1000 mm^2^. A ZwickRoell (Ulm, Germany) loading setup of the 50 kN capacity is used for the tests (Figure 6b). Additionally, Figure 6b shows the direction of loading relative to the layers during compressive testing.

The ZwickRoell testing setup is universal and can perform compression, direct tension, shear, and bending tests; therefore, it has also been used for determining the other properties presented in the next paragraphs for both the traditionally formed and printed samples. During the tests, articulations are used in the upper loading head to ensure uniform loading of the specimens and to eliminate unwanted stresses due to off-axis loading. This test approach allows for the determination of the maximum compressive, tensile, and shear stress of the material during static tests. In the case of tensile and shear tests, it also provides the possibility of assessing the adhesion of printed layers. A loading approach is adopted from the previous author’s studies.

The bending strength of the traditionally formed samples is tested on a small beam with a cross-section of 50 × 50 mm^2^ and a length of 200 mm. The test is carried out according to three-point bending with a support span of 125 mm and the load increasing at a rate of 100 N/s. Figure 7 shows the test stands.

For the printed samples, the bending strength is tested in two directions. The bent specimens have cross-sections of approximately 1000 mm^2^ and lengths of 160 mm (Figure 7b,d). The loading direction following the direction of printing is marked as ‘Z’. The loading direction perpendicular to the direction of layer printing is marked as ‘Y’ (Figure 7d). Due to the irregular cross-sectional shape of the test specimens, a simplifying assumption has been made. Rectangular cross-sections with the best possible match to the true shape are assumed for the bending strength calculations. The span of the supports is 100 mm, and the specimens are tested under a load of 100 N/s.

**Figure 8:**
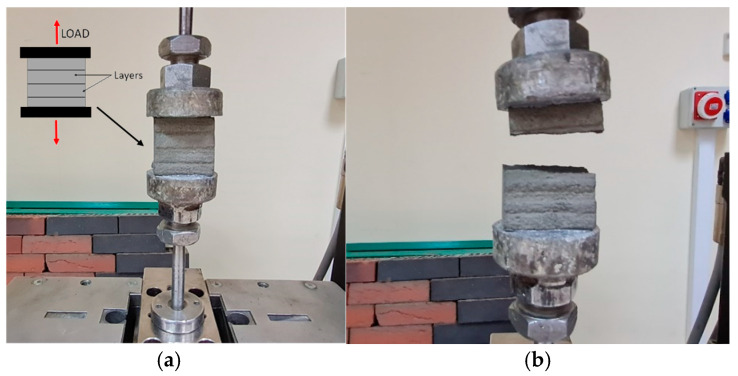
Tensile strength test for printed samples: (**a**) holding the sample for testing, (**b**) sample after testing.

The quality of the connections between the printed layers is assessed by direct tensile and shear strength tests. The method for implementing the tensile test is shown in Figure 8. The tensile test specimens are cut from larger beams. The cross-section of the samples is approximately 40 mm × 20 mm, and their height is 40 mm. The sample is subjected to tensile force according to the direction of layer printing, as shown in Figure 8a. The specimens are glued to the surface of the metal plates (steel casts with a diameter of 50 mm) through which the material is tested. The two-component glue used has a higher adhesion to the metal casts than the tensile strength of the test material. For this reason, it was possible to carry out the test. The force increment while testing is 100 N/s.

**Figure 9:**
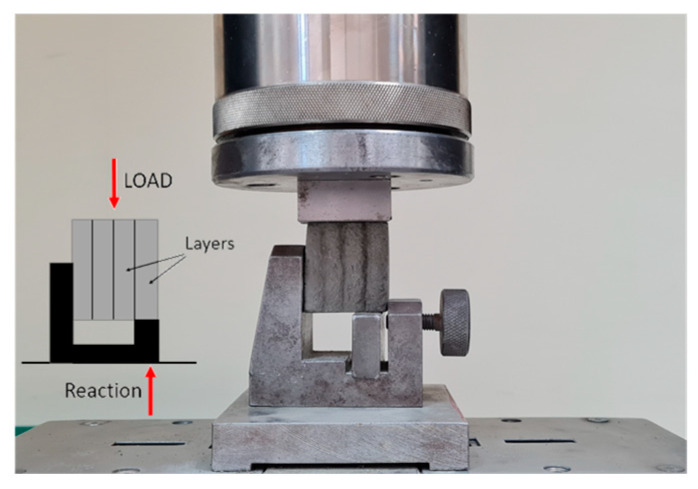
Shear strength testing of printed samples and the direction of loading in relation to the layers.

Obtaining shear stresses at the interface between the layers of the element requires a suitable support for the specimen. An external holder, shown in Figure 9, is used for this purpose. The chosen test approach facilitates the identification of maximum shear stresses and the assessment of the quality of layer adhesion within the printed material. The load increment for shear is 50 N/s.

## 3. Results and Discussion

**Figure 10:**
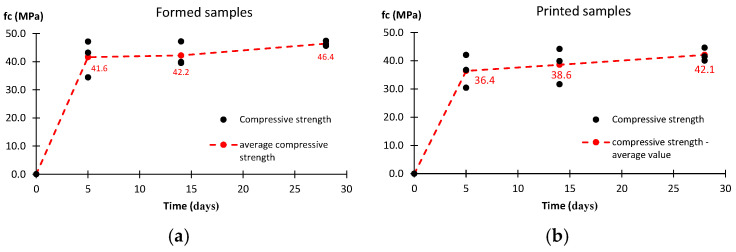
Compressive strength results for: (**a**) formed samples, (**b**) printed samples.

The test results of the compressive strength for the formed and printed specimens are shown in Figure 10. The strength development of the material is analyzed based on tests carried out after 5, 14, and 28 days. Three samples are tested each time. The black points on the graph represent a single measurement, and the red points show the average value. The observed kinetics of the strength increase indicate that the most intense strength development occurs during the first five days. The strength increase between 5 and 28 days is about 15%. The average compressive strength after 28 days for the formed samples is 46.4 MPa, and for the printed samples, it is 42.1 MPa. The results for the formed material are just 10% higher compared to the printed material.

**Figure 11:**
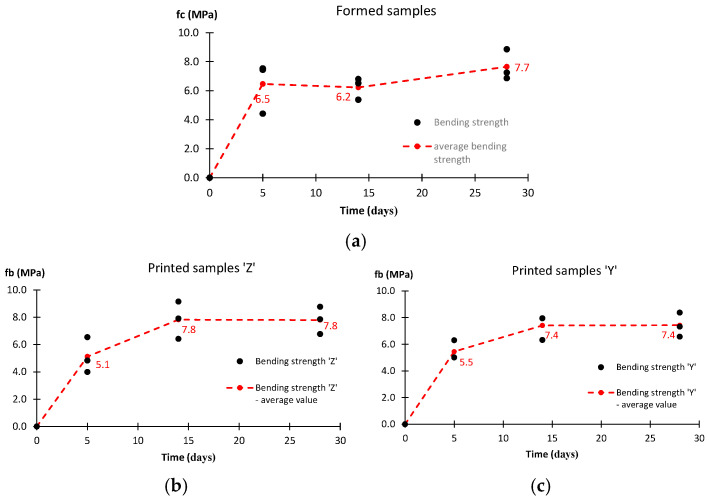
Bending strength results: (**a**) formed samples, (**b**,**c**) printed samples with load in various directions.

The results of the bending strength for different ways of producing samples are shown in Figure 11. For printed samples, the analysis is carried out in two directions. The course of the observed strength development is very similar. After 5 days, the material formed in molds has already achieved approximately 85% of the 28-day strength. Additionally, for the printed materials, the greatest increase in strength is observed in the first five days, but the strength is slightly lower. However, in the later period, the increment is greater. After 28 days, the average bending strength value regardless of the bending direction and forming method is in the range of 7.4 MPa to 7.8 MPa.

It is possible to assess the strength of the interlayer connections in a printed element by direct tension or shear tests. The direct tensile method seems to be more reliable. It allows for analysis of the quality of the contact zone when only tensile stresses are applied. The test result, while keeping the axial alignment of the specimen during the test, has a lower risk of being influenced by external factors compared to indirect methods. In the case of a lack of established procedures for testing the strength of interlayer connections in printed mineral materials, quality assessment based on static testing appears to be an appropriate approach. The choice of tests should consider the target type of loads to which the element is subjected.

**Figure 12:**
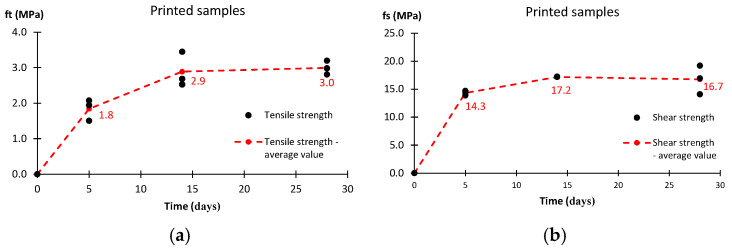
Test results for printed samples: (**a**) tensile strength, (**b**) shear strength.

The average tensile strengths for printed sample *f_t_* after 5, 14, and 28 days are 1.8, 2.9, and 3.0 MPa, respectively. All values obtained are shown in Figure 12a. During the tests, the failure of the specimens is observed at the connection zone of the layers where the minimum cross-section is measured. For shear strength *f_s_* (Figure 12b), the average strength value after 5 days is 14 MPa. Further measurements after 14 and 28 days indicate not very large changes. The increase in the average value is less than 20%. Similarly, in this case, the failure occurred at the interface between the layers of printed material.

All the strength cases analyzed show a similar development of material strength. Based on the completed range of tests, it is possible to determine three time intervals. Stage I is the first 5 days after printing the sample. After this step, the material reaches approximately 85% of the 28-day compressive and shear strengths. For the bending and tensile strengths, it is at least 60%. This is the stage of the most intense strength growth. Stage II, the period between days 5 and 14, is the stage at which the rate of strength increase slows down. A comparison of the strength on day 5 and day 14 shows a gain of about 10–20% for the compressive and shear strengths. Larger increments are observed for the bending and tensile strengths. The last stage is the period between days 14 and 28. Here, a stabilization of the mechanical properties can be reported. The observed changes do not exceed a few percent. Such observations lead to the conclusion that the strength of the tested material is mainly developed during the first 14 days. The results obtained for the compressive strength show slightly higher values for the formed samples compared to the printed samples. After 5 days, the differences are close to 15%, and after 14 days and 28 days, they are about 10%. For the bending strength, the differences are similar. After 28 days, they do not exceed more than 5%.

Similar results to those obtained in this monograph were also obtained by other researchers. For example, Nematollahi et al. [59] reported that the tensile strength of 3D-printed fly ash-based geopolymer samples without fiber reinforcement was approximately 3.5 MPa, and for samples with the 0.25% addition of polypropylene fibers, it was 5.0 MPa. This study also found that the flexural strength of 3D-printed geopolymers without fiber reinforcement was approximately 4.0 MPa and 6.5 MPa for samples with fiber addition.

Bong et al. [71] investigated 3D-printed geopolymers based on fly ash and granulated ground blast furnace slag. The compressive strength of 3D-printed geopolymers after 3 days was found to be in the range of 8.5–16.6 MPa; after 7 days, it was in the range of 13.1–21.1 MPa, and after 28 days, it was in the range of 19.8–34 MPa. Additionally, the same study reported that the flexural strength and the tensile strength of 3D-printed geopolymers cured at ambient temperature were approximately 5.5 MPa and 4.2 MPa, respectively, with variations depending on the specific curing conditions and mix designs. A 28-day interlayer bond strength of 2.7 MPa was achieved by Bong et al. [71].

Marczyk et al. [64] focused on obtaining 3D-printed concrete–geopolymer hybrids reinforced with aramid roving. This research highlighted that the compressive strength of 3D-printed geopolymer samples was generally lower than that of cast specimens, with values around 22 MPa due to weak inter-layer bonding. The tests also showed that the addition of aramid nonwoven fabric resulted in a reduction in compressive strength by approximately 30% compared to samples printed without reinforcement.

The printed elements are made in layers. A particularly sensitive area is where each layer joins together. The bonding zones have the highest risk of defects appearing, which can negatively impact the material’s strength. The similar strength values determined on the formed and printed samples indicate effective bonding of the layers. The interlayer contact zone developed in the printed material provides continuity in the material structure and causes no significant drop in the strength of the manufactured element. This confirms that the printing technology used allows for the effective use of the material’s potential.

The obtained tensile and shear strength results confirm the increase in strength of the material and interlayer connections over time. Both methods, due to the possibility of inducing a failure path through the layer contact zone, can help to assess the quality of connections in printed elements. The direct tensile method, due to the need to glue the heads and clean them after the test, is more time-consuming. In contrast, the presented shear test may be at risk of higher uncertainty due to the inaccuracy of the support used. The method of specimen positioning used during the test may not sufficiently represent pure shear conditions. It is unreasonable to assess the strength of the interlayer connections by comparing the stress values obtained from direct tension with the shear results. In each case, we are dealing with a different type of stress that dominates throughout the test and deciding about the failure of the sample. In the authors’ opinion, the choice of test used to evaluate such connections should be justified by indicating the target stresses to which the element will be subjected.

At this point, it should also be emphasized that the strength of the interlayer connections may strongly depend on the printing technology and the ensuring of optimal timing of successive layers at the component manufacturing stage.

## 4. Conclusions

In this work, the authors examine the possibility of using geopolymer binders in 3D printing technology as an additive manufacturing alternative in the construction industry. The obtained results show a significant increase in the strength of the tested samples, both formed and printed, within the first 14 days, but it is worth emphasizing that the most intense increase in the tested properties is observed during the first five days. After 28 days, the compressive strength reached 46.4 MPa for formed samples and approximately 42.1 MPa for printed samples. Analyzing the results obtained for the bending and shear strengths, a similar trend can be observed, where their intensive increase is observed until the 14th day, and between the 14th and 28th day, there is a stabilization of the tested mechanical properties. The comparative analysis of the results obtained for printed and formed samples allows us to conclude that the samples made using 3D printing technology show an effective connection between the layers, which ensures their structural integrity.

Despite the small differences in the strength properties of the casted and printed samples, this research confirms that the use of 3D printing technology allows for the effective use of geopolymer binder materials based on copper slag, which opens the door to sustainable alternatives in construction practices. Further research along with the process of improving both the material and the printing technology itself can potentially lead to solving environmental problems, revolutionizing the construction industry.

## Figures and Tables

**Table 1:** Results of the XRF analysis of the oxide composition of the mix based on Koranel copper slag.

Oxide	Result [%]	3-Sigma	Line	Int. (cps/uA)
Fe_2_O_3_	51.80	0.041	FeKa	5019.7052
SiO_2_	27.29	0.090	SiKa	42.4861
Al_2_O_3_	7.37	0.061	AlKa	5.4374
ZnO	4.49	0.012	ZnKa	422.9169
CaO	3.12	0.012	CaKa	28.2141
Cr_2_O_3_	1.42	0.007	CrKa	127.3691
MnO	0.79	0.004	MnKa	75.1951
CuO	0.73	0.005	CuKa	58.9705
TiO_2_	0.63	0.006	TiKa	26.2906
SO_3_	0.61	0.059	SKa	0.1032
P_2_O_5_	0.55	0.013	PKa	1.4194
MgO	0.52	0.060	MgKa	0.1024
K_2_O	0.24	0.005	KKa	0.6046
LOI	0.50	-	-	-

**Table 2:** Particle size distribution data for copper slag.

Parameter	Value	Standard Deviation
Mean size [μm]	5.34	0.019
D_10_ [μm]	0.26	0.002
D_50_ [μm]	4.33	0.013
D_90_ [μm]	10.44	0.040
Span	2.35	0.008

## Data Availability

The original contributions presented in the study are included in the article, further inquiries can be directed to the corresponding author.
